# A systematic review of the drug-drug interaction between Statins and Quinolones

**DOI:** 10.1186/s40360-024-00760-8

**Published:** 2024-07-10

**Authors:** Jifang Zhou, Lixia Yu, Huimin Xu

**Affiliations:** 1 Department of Pharmacy, First People’s Hospital of Linping District, Hangzhou, China; 2 Department of Pharmacy, Yuecheng District People’s Hospital of Shaoxing, Shaoxing, China; 3 Department of Pharmacy, The Second Affiliated Hospital of Medical College of Zhejiang University, Hangzhou, China

**Keywords:** Statins, Quinolones, Drug interaction, Rhabdomyolysis

## Abstract

**Background:**

Statins are widely used in cardiovascular disease (CVD) as a common lipid-lowering drug, while quinolones are widely used for the treatment of infectious diseases. It is common to see CVD in combination with infectious diseases, therefore it is often the case that statins and quinolones are used in combination. Data suggest combinations of statin and quinolone may be associated with potentially life-threatening myopathy, rhabdomyolysis and acute hepatitis. This systematic review aims to characterize data regarding patients affected by the statin-quinolone interaction.

**Methods:**

The purpose of this systematic review was to collect and evaluate the evidence surrounding statin-quinolone drug interactions and to discuss related risk mitigation strategies. The following databases were searched: PubMed (Medline), Embase, Scopus, and Cochrane Library. The systematic electronic literature search was conducted with the following search terms. In this study, three types of search terms were used: statins-related terms, quinolones-related terms, and drug interactions-related terms.

**Results:**

There were 16 case reports that met the criteria for qualitative analysis. Patients were involved in the following adverse reactions: rhabdomyolysis (*n* = 12), acute hepatitis (*n* = 1), muscle weakness (*n* = 1), hip tendinopathy (*n* = 1), or myopathy (*n* = 1). In the included literature, patients vary in the dose and type of statins they take, including simvastatin (*n* = 10) at a dose range of 20–80 mg/d and atorvastatin (*n* = 4) at a dose of 80 mg/d. There were 2 patients with unspecified statin doses, separately using simvastatin and atorvastatin. The quinolones in combination were ciprofloxacin (*n* = 9) at a dose range of 800–1500 mg/d, levofloxacin (*n* = 6) at a dose range of 250–1000 mg/d, and norfloxacin (*n* = 1) in an unspecified dose range. 81% of the case patients were over 60 years of age, and about 1/3 had kidney-related diseases such as diabetic nephropathy, post-transplantation, and severe glomerulonephritis. Nearly two-third of the patients were on concomitant cytochrome P450 3A4 (CYP3A4) inhibitors, P-glycoprotein (P-gp) inhibitors, or organic anion transporting polypeptide 1B1 (OATP1B1) inhibitors.

**Conclusion:**

Patients treated with statin-quinolone combination should be monitored more closely for changes in aspartate aminotransferase or creatine kinase (CK) levels, and muscle symptoms, especially in patients with ciprofloxacin or levofloxacin, with simvastatin and high-dose atorvastatin, over 60 years of age, with kidney-related diseases, and on concomitant CYP3A4 inhibitors.

**Supplementary Information:**

The online version contains supplementary material available at 10.1186/s40360-024-00760-8.

## Background

Hydroxymethylglutaryl coenzyme A (HMG-CoA) reductase inhibitors, also known as statins, represent widely prescribed drugs currently available for the reduction of low-density lipoprotein cholesterol, which are widely used as the mainstay therapy for the management of dyslipidaemia, including primary and secondary prevention of cardio-and cerebro-vascular disease [1]. It is estimated that around 200 million people worldwide are taking statins, which makes up 3% of the global population [2]. Although considered efficacious and safe, statins are associated with adverse effects, such as skeletal muscle toxicity and hepatic adverse reactions [3]. Globally, between 5.9 and 7.0% (depending on the diagnostic criteria used) of statin-treated patients experience symptoms of intolerance [4, 5]. Risk factors for statin-associated adverse drug reaction include advanced age (especially > 80 years, more common in women), thinness, frailty, multisystem disease (e.g., chronic renal insufficiency, especially due to diabetes), combination of multiple medications, perioperative period, combination of special medications and diet, and excessive statin doses. Certain medications increase the risk, including colchicine, verapamil, diltiazem, fibrates, protease inhibitors, and azoles [6]. So among all risk factors, drug-drug interactions play an important role and deserve further study.

Quinolones are widely used for the treatment of infectious diseases (e.g., respiratory tract infections, urinary tract infections, bacterial prostatitis, skin and other soft tissue infections, bone and joint infections, gastrointestinal infections) [7]. Adverse reactions of quinolones have certain commonalities, most commonly tendon and joint pain, and some degree of hepatotoxicity [8]. It is common to see CVD in combination with infectious diseases, therefore it is often the case that statins and quinolones are used in combination. Data suggest statin and quinolone combination may be associated with potentially life-threatening myopathy, rhabdomyolysis and acute hepatitis.

This systematic review aims to characterize data regarding patients affected by the statin-quinolone interaction, as well as describe potential etiologies, clinical ramifications, and risk-mitigation strategies associated with the drug-drug interaction between statins and quinolones.

## Methods

### Data sources and searches

We conducted a literature search following the Preferred Reporting Items for Systematic Reviews and Meta-Analyses (PRISMA) guidelines for systematic reviews. A literature search of the following databases was performed: PubMed (Medline), Embase, Scopus, and Cochrane library. We searched for all synonyms including “statins”, “quinolones” and “drug interactions”, and every statin and quinolone drug name. Search terms used across all databases included all synonyms for “statins”, “quinolones” and “drug interactions”, and every statin and quinolone drug name (for the complete search strategy, see Attachment 1), but filters varied depending on the database utilized. All databases were searched for literature up to the end of October 2022. Only articles reporting on original data, including non randomized, randomized studies, observational cohort studies, case series or case reports in adult patients aged 18 years and older were eligible for inclusion.

Exclusion criteria were (1) studies without concurrent statin-quinolone therapy, (2) studies that did not involve statin-quinolone interactions, (3) incomplete outcome reports, and (4) duplicate articles.

This study was reviewed and approved by the Medical Ethics Committee of the First People’s Hospital of Linping District, (approval number: Linping First People’s Hospital Ethics 2022 Paper No. 49).

### Data charting and evidences synthesis

In order to map the evidence, the PRISMA template was adapted. We collected data from two authors (JF and LX), then we resolved chart conflicts from a third author (HM). From each study, extract the following information: author (year), study design, patient demographics and comorbidities, statin regimen, statin intensity, quinolones regimen, quinolones dose, quinlones indication, concomitant CYP3A4 inhibitors, concomitant P-gp inhibitors, concomitant OATP1B1 inhibitors, time from quinolones initiation to onset of adverse drug reaction(ADR), hospitalization or not, ADR developed, tests for diagnostic purposes, laboratory examination, presentation, treatment and regression of ADR, time to improvement of symptoms, time to normalization of laboratory indicators, outcomes of patients, and mortality after adverse drug event.

**Fig. 1:**
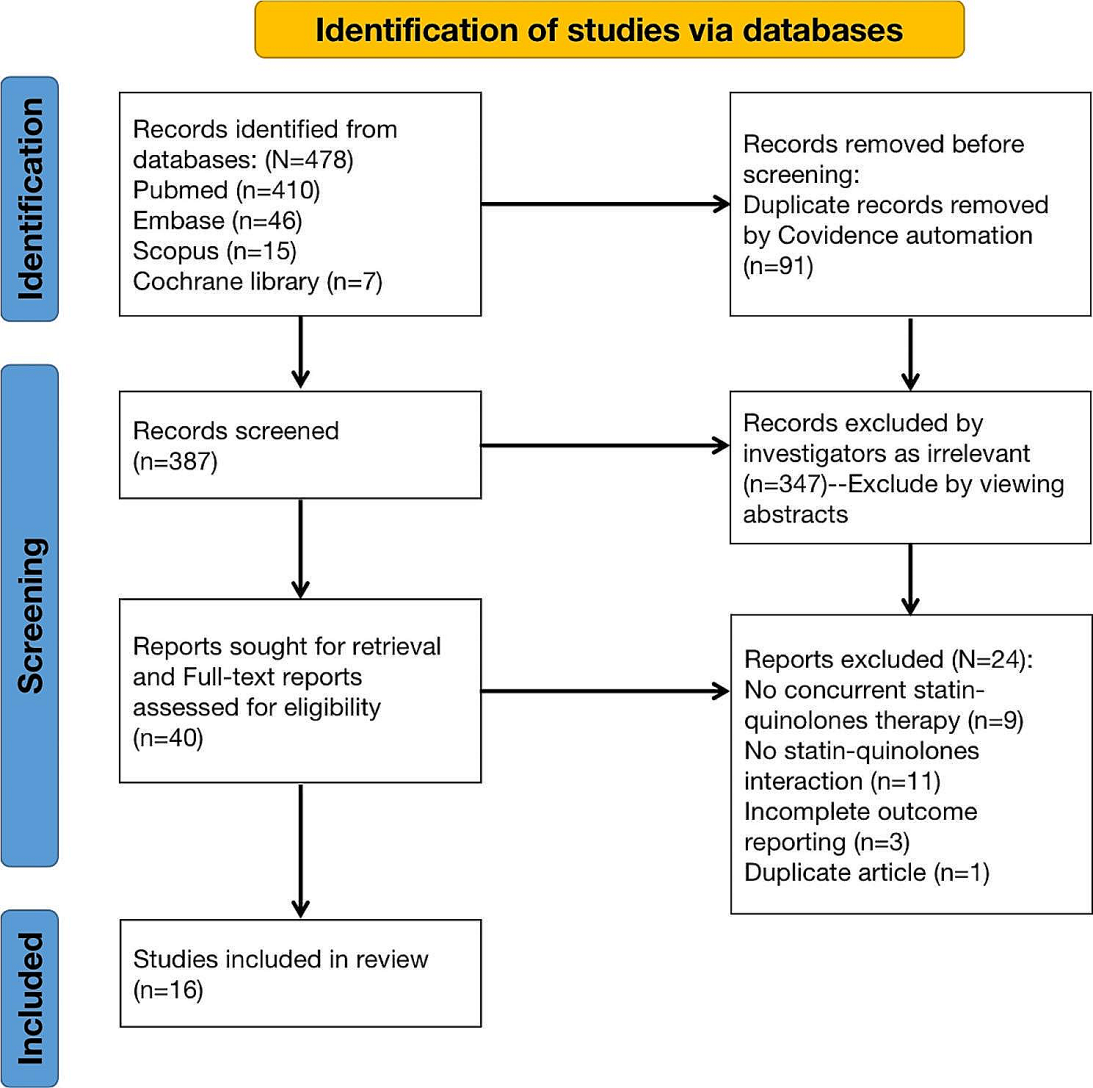
PRISMA flow diagram for Statin-Quinolones drug–drug interaction database searching of records

**Table 1:** Analysis of risk factors associated with case reports of statin-quinolone drug interactions

Author (year)	Study design	Patient demographics and comorbidities	Statin regimen	Statin intensity	Quinolones regimen	Quinolones indication	Concomitant CYP3A4 inhibitor(s)	Concomitant *P*-gp inhibitor(s)	Concomitant OATP1B1 inhibitor(s)	Whether or not statin gene analysis
Andrea (2013) [9]	Case report	64-year-old-M PMH: non-ST elevation myocardial infarction	Atorvastatin orally 80 mg once daily [duration of treatment not stated]	High	Ciprofloxacin 400 mg q12h (increased to 400 mg q8h on day 16) [route of treatment not stated]	Ventilator-associated pneumonia	None	None	None	None
Ankur (2012) [10]	Case report	77-year-old F PMH: diabetic nephropathy, hypertension, dyslipidemia, coronary artery disease, congestive heart failure	Atorvastatin orally [dosage and duration of treatment not stated]	/	Levofloxacin 750 mg intravenously and 500 mg levofloxacin orally every 48 h for 4 days	Left-sided lobar pneumonia	Amiodarone, Amlodipine	Amiodarone	None	None
Asher (2006) [11]	Case report	68-year-old M PMH: ischaemic heart disease, recurrent gouty arthritis, after kidney transplant	Simvastatin orally 40 mg once daily [duration of treatment not stated]	Moderate	Levofloxacin orally 250 mg once daily for 10 days	Consolidation of the middle lobe of the right lung	Cyclosporin A	Cyclosporin A	Cyclosporin A	None
Corrine (2012) [12]	Case report	58-year-old M PMH: hypertension, hyperlipidemia, obesity, recurrent otitis media	Simvastatinorally 20 mg once daily for 6 months	Moderate	Levofloxacin750 mg once daily for 5 days [route of treatment not stated]	Otitis media and cellulitis of his left ear	None	None	None	None
Darnis (2011) [13]	Case report	74-year-old F PMH: none	Simvastatin orally for 11 years [dosage, route not stated]	/	Norfloxacin [dosage, route and duration of treatment not stated]	Cystitis	None	None	None	None
Dorothee (2008) [14]	Case report	62-year-old M PMH: hypertension, after kidney transplant	Simvastatin orally 40 mg once daily [duration of treatment not stated]	Moderate	Ciprofloxacin 500 mg orally twice daily for 3 months	Requi infection	Cyclosporin A, Clarithromycin	Cyclosporin A, Clarithromycin	Ciclosporin A	None
Emily (2020) [15]	Case report	55-year-old M PMH: type 2 diabetes, ST-elevation myocardial infarction	Atorvastatin orally 80 mg once daily (initiated on admission)	High	Ciprofloxacin 750 mg orally twice daily (first dose starts on day 14 of hospitalisation)	Klebsiella oxytoca ventilator-associated pneumonia (VAP)	Amiodarone	Amiodarone	None	None
Farhana (2020) [16]	Case report	65-year-old F PMH: coronary artery disease	Atorvastatin orally 80 mg once daily [duration of treatment not stated]	High	Ciprofloxacin 500 mg twice daily for 5 days [route of treatment not stated]	Urinary complains	Ticagrelor	Ticagrelor	None	None
Figueira (2010) [17]	Case report	77-year-old M PMH: chronic bronchitis, arterial hypertension, hypercholesterolemia, benign prostatic hyperplasia	Simvastatin orally 20 mg once daily for 2 years	Moderate	Levofloxacin 500 mg once daily for 7 days [route of treatment not stated]	Pneumonia	Amiodarone, Amlodipine	Amiodarone	None	None
Frase r(2016) [18]	Case report	62-year-old F PMH: previous myocardial infarction, recurrent urinary tract infection	Simvastatin orally 40 mg once daily for 13 years	Moderate	Ciprofloxacin 500 mg twice daily for 4 days [route of treatment not stated]	Urinary tract infection	None	None	None	None
Jeannette (2019) [19]	Case report	65-year-old M PMH: coronary artery disease with cardiac stenting, angina, hypertension, hyperlipidemia, congestive heart failure, osteoarthritis, discitis, gout, seizures, depression, anxiety	Atorvastatin orally 80 mg once daily more than 12 months	High	Levofloxacin 750 mg orally once daily [duration of treatment not stated]	A prosthetic joint infection	None	None	None	None
Klinik (2016) [20]	Case report	73-year-old M PMH: after kidney transplant, osteoarthritis of the hip joint, heart failure, peripheral arterial occlusive disease, colonic diverticulosis hypertension, hypercholesterolemia	Simvastatin orally 40 mg once daily [duration of treatment not stated]	Moderate	Ciprofloxacin treatment for one day [dosage and route of treatment not stated]	Diarrhea	Amlodipine, Cyclosporine	Cyclosporine	Cyclosporine	None
Maria (2014) [21]	Case report	70-year-old M PMH: history of coronary artery disease, hypertension, hyperlipidemia, atrial fibrillation	Simvastatin orally 80 mg once daily for 20 days	High	Levofloxacin 500 mg intravenously once daily / 500 mg twice daily [duration of treatment not stated]	Community acquired pneumonia	None	None	None	None
Mário Bibi (2021) [22]	Case report	67-year-old F PMH: type 2 diabetes mellitus, essential arterial hypertension, dyslipidaemia, atrial fibrillation	Simvastatin orally 40 mg daily for 2 years	Moderate	Ciprofloxacin 500 mg twice daily for 7 days [route of treatment not stated]	Skin infection	Clarithromycin	Clarithromycin	Clarithromycin	None
Nicolas (2015) [23]	Case report	41-year-old F PMH: systemic lupus erythematosus complicated by severe glomerulonephritis	Simvastatin orally 20 mg once daily for 12 months	Moderate	Ciprofloxacin 500 mg twice daily for at least 5 days [route of treatment not stated]	Peritoneal dialysis-related peritonitis	Amiodarone	Amiodarone	None	Yes
Sawant (2009) [24]	Case report	77-year-old M PMH: a coronary artery bypass operation 11 years earlier, hypertension, hypothyroidism	Simvastatin orally 40 mg once daliy for 7 years	Moderate	Ciprofloxacin (2 doses) [dosage, route and duration of treatment not stated]	Urinary tract infection	Amlodipine	None	None	None

*Note* M: Male; F: Female; PMH: Previous Medical History; (CYP3A4) inhibitors: Cytochrome P450 3A4 inhibitors; P-gp inhibitors: P-glycoprotein inhibitors; OATP1B1 inhibitorsor: organic anion transporting polypeptide 1B1 inhibitors

**Table 2:** Presentation, treatment and recovery of ADRs in case reports of statin-quinolone interactions

Author (year)	Time from quinolones initiation to onset of ADR	Hospitalization	ADR developed	Tests for diagnostic purposes	Laboratory examination	Presentation of ADR	Treatment of ADR	Regression of ADR	Time to improvement of symptoms	Time to normalization of laboratory indicators	Outcomes of patients	Mortality after adverse drug event
Andrea (2013) [9]	7 days	Yes	Rhabdomyolysis	Routine laboratory exams	CK:33,687 U/L	(1) CK level increased; (2) Renal function decreased	(1) Discontinuation of atorvastatin and ciprofloxacin; (2) Dialysis	(1) CK level declined; (2) Renal function increased	5 days	Unspecified	Improved	No
Ankur (2012) [10]	2 days	Yes	Rhabdomyolysis	Routine laboratory exams	CK:7557 U/L	(1) Increasing myalgias and fatigu; (2) Diffuse musculartenderness; (3) CK level increased	Discontinuation of levofloxacin	(1) Clinical symptoms resolved; (2) CK was normalized	3–5 days	7 days	Improved	No
Asher (2006) [11]	10 days	Yes	Rhabdomyolysis	Routine laboratory exams	CK:6200 U/L	(1) Worsening myalgia and difficulty in walking; (2) Myoglobinuria was present; (3) CK level increased	(1) Discontinuation of levofloxacin and simvastatin; (2) Intravenous hydration was maintained for 5 days	(1) Muscle pain and tenderness disappeared; (2) CK level return to normal	14 days	Unspecified	Improved	No
Corrine (2012) [12]	10 days	Yes	Tendinopathy of the hip	Computed tomography	None	New-onset left lateral hip pain	Discontinuation of simvastatin	(1) Pain was improved at a 10-day recheck; (2) Reintroduction of simvastatin in 6 weeks later	10 days	Not mentioned	Improved	No
Darnis (2011) [13]	3 days	Yes	Muscular deficit and major rhabdomyolysis	(1) Creatine phosphokinase level: (2) Muscle biopsy	None	Muscular necrosis	Discontinuation of simvastatin	Not mentioned	Unspecified	Unspecified	Improved	No
Dorothee (2008) [14]	3 months	Yes	Myopathy	Electromyography	CK:2976 U/L	(1) Subacute bilateral proximal paraparesis; (2) CK level increased; (3) Decreased creatinine clearance(30 ml/min)	Discontinuation of simvastatin	(1) Muscle strength improved; (2) CK level returned to normal within 1 week; (3) Creatinine clearance increased(44 ml/min)	7 days	7 days	Improved	No
Emily (2020) [15]	4 days	Yes	Rhabdomyolysis	Routine laboratory exams	CK:48,644 U/L; LD:1382 U/L	(1) The urine was dark amber in colour; (2) CK level increased	Conversion of atorvastatin to pravastatin 10 mg daily	CK level returned to normal	Not mentioned	2 days	Improved	No
Farhana (2020) [16]	1 day	Yes	Muscle weakness	Routine laboratory exams	CK:183 U/L	(1) Extreme fatigue; (2) Progressing muscle weakness; (3) Agitation and insomnia; (4) CK level increased	Discontinuation of atorvastatin and ciprofloxacin	(1) Muscle weakness resolved; (2) CK level returned to normal in two weeks; (3) Atorvastatin was restarted after a month	21 days	Unspecified	Improved	No
Figueira (2010) [17]	7 days	Yes	Acute hepatitis	Routine laboratory exams	AST: 329 U/L, ALT:953 U/L	Elevation of transaminases	Discontinuation of levofloxacin	(1) Transaminase values in progressive decline; (2) Reintroduction of simvastatin 20 mg in 6 months later	28 days	28days	Improved	No
Fraser (2016) [18]	4 days	Yes	Rhabdomyolysis	Routine laboratory exams	CK:24,514 U/L; AST:870 U/L, ALT:240 U/L	(1) A 15-dayhistory of slowly progressing muscle weakness; (2) A 10-day history of dark brown, frothy urine; (3) Liver function disorders; (4) CK level increased	(1) Discontinuation of simvastatin; (2) Management with intravenous crystalloid fluids and urinary catheterisation	(1) Muscle weakness improve; (2) Liver function improved; (3) CK level improved	7 days	7 days	Improved	No
Jeannette (2019) [19]	19 days	Yes	Rhabdomyolysis	Routine laboratory exams	CK:11,609 U/L; AST:1613 IU/L	(1) Muscle pain and weakness; (2) CK level increased	(1) Discontinuation of levofloxacin and atorvastatin; (2) Intravenous saline injection at 150 ml/h	(1) Relevant laboratory values were back to baseline 1 week following hospital discharge; (2) Atorvastatin was resumed several months later	Unspecified	Unspecified	Improved	No
Klinik (2016) [20]	Several days (Not specified)	Yes	Rhabdomyolysis	Routine laboratory exams	CK:10,137 U/L; AST:279 U/L; ALT:87 U/L; Tn:1301 ng/L; LD:525 U/L; SCR:164 µmol/L; CK-MB:137U/L; MB:16,228 µg/L	(1) Muscle weakness of upper and lower limbs; (2) CK level increased	(1) No mention of discontinuing the medication; (2) Force diuresis with 3–4 L NaCl 0.9% daily and start intravenous furosemide Alkalinize the urine with sodium bicarbonate	(1) Increased muscle strength; (2) CK returned to normal after one week	7 days	7 days	Improved	No
Maria (2014) [21]	5 days	Yes	Rhabdomyolysis	(1) Routine laboratory exams; (2) Physical examination	CK:159,450 U/L; AST:1408 U/L	(1) Increased bilateral legs and arms weakness; (2) CK level increased	(1) Discontinuation of levofloxacin and simvastatin; (2) Treated with intravenous crystalloid hypotonic solution (100mL/h); (3) Urine alkalinization; (4) Physical therapy	(1) Symptoms improved significantly and muscle and liver enzymes normalized; (2) Normal laboratory parameters; (3) Resumption of simvastatin at 40 mg/day	A few days	A few days	Improved	No
Mário Bibi (2021) [22]	7 days	Yes	Rhabdomyolysis	(1) Routine laboratory exams; (2) Electromyography	CK:17,830 U/L	(1) Generalized muscular weakness; (2) CK level increased	(1) Discontinuation of levofloxacin and ciprofloxacin; (2) Fluid infusion	(1) Muscle strength and electromyography showed a return to normal after 6 months; (2) CK returned to normal; (3) Restarted antibiotic therapy with 400 mg moxifloxacin daily one month after admission; (4) Statins were reintroduced after antibiotic discontinuation	21 days	21 days	Improved	No
Nicolas (2015) [23]	9 days	Yes	Rhabdomyolysis	Routine laboratory exams	CK:816,000 IU/L; LD:19,200 IU/L	(1) Diffuse severe muscle pain with intense weakness; (2) The peritoneal dialysis effluent color appeared reddish; (3) Progressively anuric; (4) Severe electrolyte disorder	(1) No mention of discontinuing the medication; (2) Maintenance peritoneal dialysis treatment (3 times/day); (3) Refining the genetic analysis of statins	(1) The peritoneal dialysis effluent progressively cleared; (2) Renal function recovered; (3) Normalization of serum creatinine; (4) Correction of electrolyte disorders	Unspecified	3 days	Improved	No
Sawant (2009) [24]	1 day	Yes	Rhabdomyolysis	Routine laboratory exams	CK:28,980 U/L	(1) Severe muscle weakness and generalised muscle aches for 4 days; (2) Dark discoloration of urine; (3) CK level increased	(1) Discontinuation of ciprofloxacin and simvastatin; (2) Fluid infusion	(1) Able to walk with a Zimmer frame; (2) CK levels returned to normal	23 days	14 days	Improved	No

*Note* CK: Creatine Kinase; LD: Lactate dehydrogenase; AST: Aspartate transaminase; ALT: Alanine amiotransferase; MB: Myoglobin; Tn: Troponin; CK-MB: Creatine Kinase, MB Form; Scr: Serum creatinine

Both the time to improvement of symptoms and the time to normalization of laboratory indicators were calculated from the time the measures were taken

## Results

The search retrieved 478 records, of which 387 were screened by investigators. The specific reasons for exclusion were as follows: (1) duplicate records (*n* = 91); (2) Irrelevant when found by the investigator reading the abstract (*n* = 347); (3) Reports which were excluded by full-text search (*N* = 24): no concurrent statin-quinolones therapy (*n* = 9); no statin-quinolones interactions (*n* = 11); incomplete outcome reports (*n* = 3); duplicate articles (*n* = 1). Figure 1 depicts the PRISMA flow diagram for the screening, and ultimately, there were 16 case reports that met the criteria for qualitative analysis (Table 1).

ADRs in the included literature were rhabdomyolysis (*n* = 12) [9–11, 13, 15, 18–24], acute hepatitis (*n* = 1) [17], muscle weakness (*n* = 1) [16], tendinopathy of hip (*n* = 1) [12], and myopathy (*n* = 1) [14] (Table 2). CK was reported in 81% (*n* = 13) of the case reports with a range between 183 and 816,000 units/L, which all above the normal value for CK [9–11, 14–16, 18–24]. All patients were given statins, including simvastatin (*n* = 10) [11–13, 17, 18, 20–24] at a dose range of 20–80 mg/day and 66.7% of the remaining patients received 80 mg/day of oral atorvastatin (*n* = 4) [9, 15–16, 19]. And there were 2 patients with unspecified statin doses, separately using simvastatin and atorvastatin. Different types of quinolone antibiotics for the patients involved, including ciprofloxacin (*n* = 9), levofloxacin (*n* = 6), and norfloxacin (*n* = 1). Among the included articles, the drug combinations which showed ADRs were simvastatin and ciprofloxacin (*n* = 6), simvastatin and levofloxacin (*n* = 4), atorvastatin and ciprofloxacin (*n* = 3), atorvastatin and levofloxacin (*n* = 2), and simvastatin and norfloxacin (*n* = 1). The dose of quinolones is heterogeneous among the studies. Seven case reports reported ciprofloxacin doses between 400 and 750 mg twice daily [9, 14–16, 18, 22, 24] and 2 case reports did not report ciprofloxacin doses [20, 24]. Six case reports reported levofloxacin doses between 250 to 1000 mg/d [10–12, 17, 19, 21]. It was unclear from 1 case report about the norfloxacin dosage [13]. In the included literature, the majority of patients’ adverse reactions occurred between 1 and 19 days of combined statin and quinolone use. Only one patient was readmitted for myopathy 3 months after the combination, but the exact timing of the ADR was not clear [14]. High-intensity statins were implicated in 5 case reports [9, 15–16, 19, 21], with four patients taking 80 mg per day of atorvastatin and one patient using 80 mg per day of simvastatin, while moderate-intensity statins in 9 case reports [11, 13, 15, 18–24]. It was unclear from two case reports about the statin dosage, one involving atorvastatin and one involving simvastatin [10, 13]. Approximately 63% of the case report patients were male [9, 11, 12, 15, 17, 19–21, 24] and 81% of the patients were over 60 years old [9–11, 13, 15–22, 24]. Most patients presented with comorbidities, with the most common being hypertension (56%, *n* = 9), coronary artery disease (56%, *n* = 9), dyslipidemia (44%, *n* = 7), diabetes (19%, *n* = 3) and renal abnormalities (31.25%, *n* = 5). Two cases identified solid organ transplant patients who were taking concomitant immunosuppressants, specifically cyclosporine [11, 14]. Ten case reports (62.5% of all studies) reported patients were taking concomitant CYP3A4, P-gp and/or OATP1B1 inhibitors. Four case reports identified patients taking with amiodarone (a concurrent CYP3A4 and P-gp inhibitor) [10, 15, 17, 23]. Four cases reported patients used amlodipine (a CYP3A4 inhibitor) [10, 17, 20, 24]. Three cases report identified patients taking concomitant cyclosporine (a CYP3A4, P-gp and OATP1B1 inhibitor) [11, 14, 20]. Two cases described patients taking clarithromycin (a CYP3A4 and P-gp inhibitor) [14, 22]. One case reported the patient with a concurrent ticagrelor (a concurrent CYP3A4 and P-gp inhibitor) [16].

All of the 16 adverse reactions reported by this systematic review required hospitalization. Seven case reports (44%) managed the ADR by discontinuing both the statin and quinolones [9, 11, 16, 19, 21–22, 24], 4 cases (25%) discontinued the statin alone [12–14, 18] and 2 case (12.5%) discontinued the quinolones alone [10, 17]. One case (6%) managed the ADR by switching to another statin [15]. Seven cases (44%) managed the ADR by hydrating with intravenous fluids [9, 16–21], and 2 case (12.5%) were treated with dialysis [9, 23], while 2 patients (12.5%) treated with alkalinised urine [20–21]. In the included literature, all cases were improved after relevant treatment. Patients’ symptoms of adverse reactions improved after 3–28 days. None of the patients experienced death as a result of the statin and quinolone combination.

## Discussion

In this systematic review, 16 patients experienced ADRs related to the combination of statins and quinolones. These reported ADRs included rhabdomyolysis (*n* = 12), acute hepatitis (*n* = 1), muscle weakness (*n* = 1), hip tendinopathy (*n* = 1) or myopathy (*n* = 1), with the vast majority (81%) of these symptoms being associated with CK elevation. The drug combinations presenting with ADRs were, in order of frequency, simvastatin and ciprofloxacin (*n* = 6), simvastatin and levofloxacin (*n* = 4), atorvastatin and ciprofloxacin (*n* = 3), atorvastatin and levofloxacin (*n* = 2), and simvastatin and norfloxacin (*n* = 1). In summarising the most common characteristics of patients affected by statin-quinolone interactions described in this systematic review, 10 subjects (62.5% of all patients) were combined with other drugs such as CYP3A4 inhibitor, P-gp inhibitor, OATP1B1 inhibitor, 13 subjects (81.25% of all patients) were over 60 years of age; 14 (88% of all studies) reported patients taking medium to high intensity statins and 5 subjects (31.25% of all patients) identified patients with comorbid renal disease. On average, ADRs occurred after 15 days of combined statin and quinolone therapy. All patients were hospitalised for the aforementioned adverse reactions, 13 patients chose to discontinue the drug, seven of them discontinuing both statin and quinolone, 4 patients discontinuing only the statin and two discontinuing only the quinolone; 7 patients used hydration, 2 patients underwent dialysis; and 1 patient adjusted the type of statin. In addition, genetic analysis was performed in only one of the 16 cases to clarify possible factors for the development of ADR. All patients improved after appropriate treatment.

Through this systematic review, we found the types of the statins and quinolones were risk factors for ADR following the combination of statins and quinolones. Statins can be grouped according to differences in enzymatic metabolism, for example simvastatin and atorvastatin are mainly metabolised by CYP3A4 isoenzymes, whereas fluvastatin and pravastatin are mainly metabolised by CYP2C9 enzymes [25]. In the included reports, the types of statins were dominated by simvastatin (*n* = 11) and atorvastatin (*n* = 5), and the types of quinolones were ciprofloxacin (*n* = 9), levofloxacin (*n* = 6) and norfloxacin (*n* = 1), which are all CYP3A4 inhibitors. Therefore, combination of statins and quinolones metabolised by CYP3A4 are at greater risk of drug interactions. Secondly, the dose of the statin was also a risk factor for ADR following the combination of statins and quinolones. In this systematic review, statin doses were mentioned in 14 of the 16 cases, all at moderate to high intensity statins. Atorvastatin had an ADR risk only at the highest dose (80 mg/d), whereas simvastatin had a risk at all doses (dose range fluctuated from 20 to 80 mg/d). However, the dose and treatment duration of quinolones may not affect the ADRs that occur after the combination of statin and quinolone. Quinolone doses were mentioned in 13 of the 16 cases, with seven cases reporting ciprofloxacin doses between 400 and 750 mg twice daily and six cases reporting levofloxacin doses between 250 and 1000 mg/day. Only 4 of the 16 cases used levofloxacin at a dose slightly above the usual dose. Quinolone treatment duration were mentioned in 9 of 16 case reports, with specific durations ranging from 4 days to 3 months. Thirdly, the combined medications were risk factors for ADR following the combination of statins and quinolones. In 16 publications, 10 patients were combined with CYP3A4 inhibitors, P-gp inhibitors, and OATP1B1 inhibitors that affect statin metabolism, such as amiodarone, amlodipine, cyclosporine, clarithromycin and ticagrelor. Amiodarone is an inhibitor of the pharmacological enzyme P450 3A4 and P-gp, which affects simvastatin and atorvastatin, with the most adverse reactions reported in combination with simvastatin. Amlodipine is known to have a competitive inhibitory effect on the metabolic activity of CYP3A4/5. Pharmacokinetic modelling has shown that 10 mg amlodipine significantly increases the bioavailability and decreases the clearance of simvastatin when it is combined with simvastatin [26, 27]. In addition, combination therapy with cyclosporine A and simvastatin increased the area under the curve of simvastatin by eightfold by competing for the drug binding site of the cytochrome P450 3A4 enzyme [28–30]. Also cyclosporine A is a potent inhibitor of P-glycoprotein and prolongs levofloxacin concentrations in tissues. Clarithromycin is an inhibitor of organic anion transporter polypeptide 1B1 (OATP1B1), a transporter involved in the metabolic pathway of all statins, including those not metabolised by CYP3A4 [31]. Ticagrelor is metabolised by cytochrome P4503A4, which, like most statins, competitively inhibits CYP3A4 isoenzymes, leading to the accumulation of statins metabolised by CYP3A4. Finally, patients with creatinine clearance below 30 ml/min and older patients were all reported increasing risks of statin-related myopathy [26–30]. In this systematic review, we also found that elderly patients and renal insufficiency are all factors that contribute to the increased risk of combining statins and quinolones. We found 13 patients (81.25% of all patients) were over 60 years of age and 5 patients (31.25% of all patients) were with combined renal insufficiency.

In general, the first step after an ADR is to stop the suspected drug. According to the relevant guidelines, the main treatment principle for rhabdomyolysis is to promote the excretion of myoglobin from the kidneys and protect renal function, which is divided into the following four options: (1) hydration: saline infusion is recommended; (2) alkalinize the urine: apply sodium bicarbonate to alkalize the urine; (3) correction of electrolyte disturbances caused by rhabdomyolysis, such as low blood calcium and high blood potassium; (4) dialysis at the appropriate time [32, 33]. Faced with ADRs from the combination of statins and quinolones, 13 patients discontinued their medication, of whom 7 discontinued both statins and quinolones, 4 discontinued only statins, 2 discontinued only quinolones and 1 adjusted the type of statin; 7 patients chose to rehydrate and 2 went on dialysis; all patients subsequently showed improvement. We found a significant effect regardless of whether statins or quinolones were discontinued. Regarding the choice of hydration medication, some chose 0.9% sodium chloride injection, some chose intravenous crystalloid, some chose intravenous crystalloid hypotonic solution, and 2 patients combined catheterisation or the diuretic furosemide as an adjunct to diuresis. In the early stages of ADR, fluid replacement, diuresis and active correction of electrolyte disturbances are the mainstays, and sodium bicarbonate can be used to alkalinise the urine. If severe kidney damage has been caused or if symptoms of oliguria or anuria develop, haemodialysis or haemofiltration treatment can be administered. In most cases, doctors were able to detect ADRs caused by the combination of statins and quinolones and treated them as recommended, and the prognosis was generally good.

## Conclusions

In this systematic review, 16 cases reported ADRs while receiving a statin-quinolone combination. The pharmacokinetic and pharmacodynamic properties of quinolones and statins, the combination of CYP3A4 isoenzymes and P-gp inhibitors and OATP1B1 inhibitors, reduced renal function and advanced age are all factors that contribute to adverse reactions following the combination of quinolones and statins. Therefore, patients treated with statin-quinolone combinations should be monitored intensively for changes in liver function and muscle enzymes and if ADRs develops, it can be reversed by timely drug discontinuation, hydration, diuresis and dialysis.

### Electronic supplementary material

Below is the link to the electronic supplementary material.

Supplementary Material 1

## Data Availability

The dataset used and/or analysed during the current study is available from the corresponding author on reasonable request.

## Abbreviations

CVD: Cardiovascular disease
CYP3A4: Cytochrome P450 3A4
P-gp: P-glycoprotein
OATP1B1: Organic anion transporting polypeptide 1B1
CK: Creatine kinase
HMG-CoA: Hydroxymethylglutaryl coenzyme A
PRISMA: Preferred Reporting Items for Systematic Reviews and Meta-Analyses
ADR: Adverse drug reaction
VAP: Ventilator-associated pneumonia
CK: Creatine Kinase
LD: Lactate dehydrogenase
AST: Aspartate transaminase
ALT: Alanine amiotransferase
MB: Myoglobin
Tn: Troponin
CK-MB: Creatine Kinase, MB Form
Scr: Serum creatinine
