# Supplementary material for: A systematic review of the drug-drug interaction between Statins and Quinolones
Source: BMC Pharmacol Toxicol. 2024 Jul 10;25:39. doi: 10.1186/s40360-024-00760-8 (PMC11234672; doi:10.1186/s40360-024-00760-8)
Supplement: Supplementary file 1 — Supplementary Material 1 [file 40360_2024_760_MOESM1_ESM.doc]

**Attachment 1**

The search strategy for PubMed is stated below. The search terms for other databases were similar.

#1 Hydroxymethylglutaryl CoA Reductase Inhibitors[MeSH Terms]

#2 Hydroxymethylglutaryl CoA Reductase Inhibitors [Title/Abstract]

#3 Atorvastatin [MeSH Terms]

#4 Rosuvastatin [MeSH Terms]

#5 Fluvastatin [MeSH Terms]

#6 Pivastatin [MeSH Terms]

#7 Pravastatin [MeSH Terms]

#8 Lovastatin [MeSH Terms]

#9 Simvastatin [MeSH Terms]

#10 1-9 /or

#11 Quinolone [MeSH Terms]

#12 Quinolone [Title/Abstract]

#13 Norfloxacin [MeSH Terms]

#14 Ofloxacin [MeSH Terms]

#15 Ciprofloxacin [MeSH Terms]

#16 Levofloxacin [MeSH Terms]

#17 Moxifloxacin [MeSH Terms]

#18 Pipemidic acid [MeSH Terms]

#19 Jimifloxacin [MeSH Terms]

#20 Floroxacin [MeSH Terms]

#21 Fluoroquinolone [MeSH Terms]

#22 Nalidixic acid [MeSH Terms]

#23 Piromidic acid [MeSH Terms]

#24 Cinoxacin [MeSH Terms]

#25 Miloxacin [MeSH Terms]

#26 Perfloxacin [MeSH Terms]

#27 Enoxacin [MeSH Terms]

#28 Gatifloxacin Quinolone [MeSH Terms]

#29 11-28/or

#30 Drug interactions [MeSH Terms]

#31 Interaction [Title/Abstract]

#32 30-31/or

#33 10 AND 29 AND 32
